# Improving health in cities through systems approaches for urban water management

**DOI:** 10.1186/s12940-016-0107-2

**Published:** 2016-03-08

**Authors:** L. C. Rietveld, J. G. Siri, I. Chakravarty, A. M. Arsénio, R. Biswas, A. Chatterjee

**Affiliations:** Delft University of Technology, Delft, The Netherlands; United Nations University International Institute for Global Health, Kuala Lumpur, Malaysia; Public Health Engineering Department, Government of West Bengal, Kolkata, India; Austrian Institute of Technology, Vienna, Austria; School of Planning and Architecture, Bhopal, India

**Keywords:** City planning, Systems theory, Urban health, Urban sanitation, Waterborne diseases, Water supply, Recreation

## Abstract

**Background:**

As human populations become more and more urban, decision-makers at all levels face new challenges related to both the scale of service provision and the increasing complexity of cities and the networks that connect them. These challenges may take on unique aspects in cities with different cultures, political and institutional frameworks, and at different levels of development, but they frequently have in common an origin in the interaction of human and environmental systems and the feedback relationships that govern their dynamic evolution. Accordingly, systems approaches are becoming recognized as critical to understanding and addressing such complex problems, including those related to human health and wellbeing. Management of water resources in and for cities is one area where such approaches hold real promise.

**Results:**

This paper seeks to summarize links between water and health in cities and outline four main elements of systems approaches: analytic methods to deal with complexity, interdisciplinarity, transdisciplinarity, and multi-scale thinking. Using case studies from a range of urban socioeconomic and regional contexts (Maputo, Mozambique; Surat and Kolkata, India; and Vienna, Austria).

**Conclusion:**

We show how the inclusion of these elements can lead to better research design, more effective policy and better outcomes.

**Electronic supplementary material:**

The online version of this article (doi:10.1186/s12940-016-0107-2) contains supplementary material, which is available to authorized users.

## Background

Water is an elemental part of the fabric of urban lives, providing sustenance and sanitation, commerce and connectivity. Our fundamental needs for water have always determined the location, size and form of our cities, just as water shapes the character and outlook of their citizens. Urban health is inextricably linked with water. From the first cities, planners have appreciated the potential linkages of water with health and the need for consistent water supplies see, e.g., [1]. Indeed, the modern field of public health owes a strong debt to the sanitary engineers who strove to provide potable water and safe disposal of human wastes in burgeoning cities of the Industrial Revolution [2].

Scientists and decision-makers have recently begun to appreciate that, as in the case of other urban systems, the linkages between water management, health and sustainability are complex in ways that undermine the effectiveness of traditional approaches [3]. Unprecedented urban populations and densities, intra-urban inequities, and inter-urban mobility pose serious new problems, and climate change adds a novel and uncharted dimension [4, 5]. This has, in some cases, led to worsening urban health, or to increased risks—for instance, some water-associated diseases like dengue are on the rise globally [6] while others, like cholera, nominally controlled in the developed countries, continue to pose serious threats elsewhere [7]; many regions face increased food and water scarcity, and many urban slums present conditions that challenge effective water management [8].

There is growing recognition in the scientific community that such complex problems are best managed using “systems approaches,” which account for the complex interrelationships between connected systems and allow for the integration of knowledge across a wide range of disciplinary and policy domains [9–12]. This paper reviews linkages between water and health and describes how the application of important elements of systems approaches (i.e., models of complexity, interdisciplinarity, transdisciplinarity, and multi-scale thinking) can yield benefits for health in the context of urban water management. For illustration, the paper draws on a set of case studies over a range of urban scales in a diverse set of geographic, economic and socio-cultural contexts, focusing on how the inclusion of these features of systems thinking have led to positive impacts on health and on the identification of co-benefits with policies for environmental sustainability. The goal of this work is to synthesize knowledge on water management, urban health and systems thinking, to illustrate how some applied interventions have made use of several of these principles, and to call for a deeper and more systematic application of such methods.

### Urban linkages between water and health

A multitude of pathways links water to health in cities. Most fundamentally, human survival depends on consistent supplies of sufficient safe water, an issue of growing concern in areas prone to climate-change-induced scarcity [4]. Water may act as a direct carrier for pathogens or toxins or a habitat for disease vectors or reservoirs [13]. These proximal influences on health can propagate downstream, influencing nutrition, physical and mental development (for example, where chronic enteric disease causes malnutrition or stunting) [14–16] and metabolic health status (as with anemia resulting from chronic intestinal bleeding associated with worm infestation) [17]. Chronic water-related illnesses may result in reduced work capacity or school absenteeism, often with long-term effects on wealth, education and quality of life (see, e.g., [18, 19]). There is also evidence that water influences mood and self-esteem, and therefore mental health [20]. Where available for recreational use, water can also affect wellbeing and physical activity, and therefore obesity and non-communicable disease [20]. Further, water management is an integral element of urban safety, for example in fire-fighting, flood control and long-term management of sea-level rise.

The most complex and intractable urban water-related health issues often arise where various systems intersect. Cross-sectoral feedbacks tend to pose particular challenges for environmental and social policy [21], both because it is more difficult for managers in one sector to visualize root causes or to appreciate the outcomes of their own actions in other sectors and because decision-making pathways fail to provide the authority needed to address emerging multi-sectoral problems. Water management systems are intimately linked with systems that address other urban needs, like agricultural production, food hygiene and sanitation, which are usually beyond the reach of water managers. For example, water often plays critical roles in the transportation of goods and people in urban areas or for urban uses, and in urban energy generation—each of which have implications for health in cities. Relationships in urban systems are often complex, involving feedbacks and cascading consequences, and the resulting health issues can be difficult to anticipate or control. For example, the 2010 Tohoku earthquake, a geological event, caused a tsunami, which caused the Fukushima reactor meltdown, which led to radiation release, with consequences for urban health at each stage [22].

### Urban water management and health

Water management plays a major role in protecting the health of urban populations worldwide. Water management systems endeavor to assure access to high-quality potable water free of contaminants and to guarantee that waste streams—greywater, wastewater and fecal sludge—are adequately conveyed and treated, in order to minimize their contact with humans and protect the environment. Water managers regulate water courses and sources, minimizing the effects of floods and droughts, and, in coastal cities, work to mitigate the risks associated with extreme weather events and long-term sea level rise. Collectively, “the availability of an acceptable quantity and quality of water for health, livelihoods, ecosystems and production, coupled with an acceptable level of water-related risks to people, environments and economies” has been termed “water security” [23], the assurance of which constitutes a major element of urban governance. Urban water management may also include the provision and management of urban “blue space,” which has benefits for physical and mental health in cities.

The challenges that face urban water management are substantially different in developed and developing countries [24]. In the former, water management systems tend to be robust and well-resourced; water quality is typically high, and access is rarely an issue [25]. Outbreaks of infectious disease are usually quickly contained, and shortages are typically transient, with people in developed countries being much less prone to suffer from water-borne diseases than inhabitants of less developed areas, for example in Africa and Southeast Asia [26]. Concerns tend to focus on the reduction of wasteful consumption and on ensuring long-term sustainability, particularly in the face of increasing temperatures, precipitation changes and sea level rises. This has been, for example, addressed by the European Environmental Agency [27], with the agency calling “for more knowledge to support a multi-level approach to urban adaptation” an idea that is central in this manuscript. Increasingly costly extreme events have also resulted in a greater focus on resilience and mitigation of water-related economic and health losses in coastal cities. Cities in the developed world also have greater resources to devote to aesthetic and recreational aspects of water use.

In developing countries, water management systems tend to face infrastructural and resource deficits, leaving cities with limited access to safe drinking water; sanitation services are also often unavailable for large fractions of the population. In sub-Saharan Africa, for example, 38 % of people do not have access to safe drinking water and 26 % practice open defecation [28]. Indeed, one of the core elements of the definition of slums—which house 800 million urban dwellers worldwide [29]—is a lack of access to safe water. Pathogen reservoirs are much larger than in the developed world, and disease vectors may breed in stagnant or unmanaged water courses. As well, less stringent or less-well-enforced environmental regulation may lead to higher ambient levels of toxins. Each of these factors poses additional challenges to already limited infrastructural systems. Moreover, increased exposure to communicable diseases in a context when non-communicable diseases are also on the rise—known as the “double burden” of disease [30]—further strains limited urban resources. Finally, developing-world cities face all the same challenges of environmental change and sustainability seen in the developed world—often more so, given that climate change is expected to disproportionately impact less-developed countries.

### Methods

A growing awareness of complexity has led to recognition of the need for systems approaches (see, e.g., [11]). Terminology and usage vary, and a full discussion of the history and theory of systems thinking as it has arisen in many fields is beyond the scope of this paper. Yet a variety of elements of such approaches have repeatedly been recognized as essential; these include (but are not necessarily limited to): analytical methods to deal with complexity; interdisciplinarity; transdisciplinarity; and multi-scale analysis [31]. We highlight these in the context of water management and health. In particular, we distinguish between *systems methods*, which are analytic approaches to evidence gathering, generally involving quantitative models of complex systems, and *systems approaches*, which are holistic efforts to resolve complex problems by understanding the systems involved and applying appropriate corrective actions.

In contrast to traditional empirical approaches which attempt to isolate causal relationships between individual variables, systems methods examine the effects of multiple actions on multiple outcomes over time, often through the application of simulation models. Accordingly, they are able to capture the feedbacks and non-linear behavior inherent in complex systems [32, 33]. Without an understanding of such phenomena, urban policies often fail to achieve desired goals over the long-term, and frequently create unanticipated and undesirable secondary effects. For example, floodwater levees reduce transient risk of flooding, but in so doing have multiple unintended consequences: they concentrate water, leading to increased risks from larger-scale floods that exceed levee specifications; they decrease public perception of risk, leading to urban development in risky areas; and they diminish the experience of urban managers with small floods. Each of these factors tends to increase vulnerability [32].

Systems approaches encompass interdisciplinarity, particularly important in the context of cities, since complex urban systems naturally span a diversity of natural and institutional domains. That is, proper understanding of the system generally requires the input of experts from a variety of academic fields and professional disciplines, intensively exchanging views, and learning from and exploring creative solutions with experts from other fields. For example, Batterman [13] outlines an interdisciplinary approach to water-based infectious diseases that encompasses “ecologic, anthropologic, engineering, political/economic, and public health fields.” This runs counter to traditional approaches wherein researchers and policy-makers operate in academic or professional siloes, attempting to address problems individually in a context where all problems are connected. A practical consequence of the genuine application of interdisciplinarity is the identification of co-benefits— i.e., ancillary benefits in one domain (e.g., urban health) arising from actions taken to resolve a problem in some other domain (e.g., climate change mitigation) [34].

Transdisciplinarity, defined as the co-production of knowledge by scientists, decision-makers and other stakeholders, including lay stakeholders and communities, is also critical. Too often, urban policy is designed on the basis of scientific or political goals that do not take into account the perceived needs of the communities in which they are applied. No matter how valid, such actions are unsustainable, and the failure to consult can engender a lack of trust which hinders further efforts to resolve new issues. Just as important, local stakeholders have access to evidence and an understanding of local needs and processes that is otherwise inaccessible to researchers. Systems approaches ideally combine the expertise of scientists, the practicality of policy-makers and the local knowledge of communities to create feasible, actionable, valuable interventions. The City Blueprint methodology [35], for example, represents an effort to develop a comprehensive (interdisciplinary) set of indicators for urban water sustainability; in providing a simple but comprehensive baseline assessment for urban water cycle services, it represents a quick and transparent way to involve (transdisciplinary) stakeholders in understanding water systems and envisioning water services.

Finally, systems approaches usually involve consideration of phenomena acting at multiple scales. Urban health depends critically on actions taken and natural systems operating outside city boundaries. Similarly, cities impact areas far beyond their own borders, including not only surrounding areas but planetary systems (e.g., climate, trade, and migration, the spread of innovation, and the dissemination and adoption of good practices). Within cities, actions are taken by institutions at many scales, and urban systems often generate inequities. Understanding such issues requires the explicit application of multi-scale thinking.

### Results and discussion

The following series of case studies highlights situations in which one or more elements of systems approaches, as outlined above, were applied. These are not intended to demonstrate the full, proactive, conscious application of systems approaches, but rather are illustrative of the benefits that can accrue from even partial inclusion of such approaches in water management strategies. They were selected to represent a range of socio-economic, geographic and urban contexts, and the intersection of water with various other urban systems.

#### Street food in Kolkata, India

In many cities, so-called “street foods” play a large and increasing role in nutrition [36]. Because such foods are generally low-cost, easily accessible, and cater to traditional tastes, they tend to be of particular importance in poor or informal communities and in areas experiencing high urban migration [37, 38].

The street food system is particularly vulnerable to deficiencies in water management. This is reflected in the fact that street vendors often have diminished infrastructural access to clean water for drinking or hygiene when compared with fixed-location providers [39]. Moreover, street vendors often lack basic education, and rarely have formal training in food hygiene (or at times basic personal hygiene) [39]. As well, given that they cater to the poor, street food stalls are frequently situated in particularly unhygienic locations [39].

In this context, a traditional view of water management would likely fail to provide effective solutions. A highly-developed street food system requires provision of potable water at curbside, non-building, often variable locations; abundant facilities for public sanitation distributed along corridors of high volume; increased frequency of street cleaning and solid waste collection; potentially re-design of sewerage, to the extent that street food consumption generates solid waste; and other municipal actions. Identifying and implementing such actions requires systems approaches.

A 1992-3 study in Kolkata (population 11.0 million, 1991; 14.1 million, 2011), supported by the Food and Agriculture Organization, highlighted both positive and negative aspects of street foods [36], and led to the “Improving Street Foods in Calcutta” program. The All India Institute of Hygiene and Public Health (AIIHPH), in collaboration with the Calcutta Municipal Corporation, surveyed local vendors and consumers, characterizing locations, food-handling processes, types of food sold and the customer base, and testing samples for microbiological contaminants. Street foods were found to offer significant benefits with respect to convenience, accessibility and employment, among others, yet a number of major problems were identified. Among these were unsafe food handling and poor personal hygiene, poor water quality, lack of hygienic solid waste disposal options, improper display and storage of food, and poor local environmental conditions. Major observed contaminants included fecal coliform, *Escherischia coli*, yeasts and molds, *Salmonella spp.*, and *Shigella spp.*

This study led to significant changes in the management of street vending in Kolkata. For example, it identified increased provision of safe potable water and continued upgrading of waste water collection and disposal systems as critical to improving health in the context of the street vending system [36]. Other recommendations encompassed activities in a wide range of sectors, including solid waste management, hygiene training, licensing and inspection of street vendors, the provision of small loans, development of improved kiosks, and upgrading of sidewalks. Beyond this systems-based approach to solutions, a critical aspect of the study and program was transdisciplinary engagement with a multitude of stakeholders, including street vendors themselves, public health and municipal authorities, scientists, and police officers, who took on an active role in oversight [36, 40]. The study has led to significant improvements in water supply, water quality and health in Kolkata.

#### Municipal waste management and co-benefits for health in Surat, India

Surat (population 1.5 million, 1994; 4.4 million, 2011) is a rapidly growing city in Gujarat, India. Long known as one of the least hygienic cities in the country, Surat experienced an outbreak of pneumonic plague in 1994 associated with flooding, poor hygiene and inadequate waste disposal; this prompted the municipal government to undertake a wide-ranging set of actions to improve the urban environment, including a transformation of the water management system [41]. By the end of the first decade of the 2000s, Surat was considered one of the cleanest cities in India.

Surat took a broadly intersectoral approach to environmental management, with significant impacts on health. Among other actions, it radically extended and improved its water supply, sanitation and drainage infrastructure, while also decentralizing administrative functions and involving the private sector in urban service provision [42]. In just a few years following the plague outbreak, diarrheal incidence in Surat dropped from nearly 50 % of all cases in Gujarat Province to just 10 % [43]. More recent surveys have shown continuing reductions in the incidence of some vector-borne diseases [44]. According to a World Bank analysis, health gains were achieved through “decentralization, improving efficiency, enhancing infrastructure performance standards, and strengthening health services” [43].

A key element of the municipal transformation was the ability to pursue multiple cross-sectoral goals simultaneously through a judicious understanding of co-benefits. For example, the Surat Municipal Corporation was the first municipal corporation in India to operate its sewage treatment plants (STPs) using biogas energy. The primary goal of this action is to recover methane for power generation, in view of climate-change concerns. Methane recovery is viewed as a “best practice” in wastewater management, because it produces a wide range of co-benefits. It generates electricity used by the STP, thus mitigating greenhouse emissions that would have resulted from reliance on the fossil-fuel-based grid power supply [44], and increases employment and public savings, while also reaping the benefits typically associated with wastewater treatment: reductions in water pollution and water-borne disease and generation of organic solids for fertilizer, among others. Direct benefits of the project include a total reduction of 80,000 tonnes of carbon dioxide equivalent emissions per year from the four STPs in Surat [41]. Through the embrace of a co-benefits framework, water management is thus contributing not only to expected reductions in water-borne disease in Surat, but also to improvement in a wider set of environmental and health outcomes.

The “better coordination across agencies and actors” prompted by the 1994 plague event even set the stage for Surat to respond to major floods in 2006 by adopting a City Resilience Strategy in 2011 and establishing a Climate Change Trust to integrate climate resilience into city development initiatives [42, 45].

#### Safe water reuse in Maputo, Mozambique

Mozambique has been termed the worst country in the world with respect to access to clean and safe water [44] and its capital, Maputo (population 1.1 million, 2007) [46] shares many of the problems of other cities in developing countries, as outlined below. Maputo can be roughly divided into the city-centre and peri-urban areas, each with very different social and architectural characteristics [47] and water management issues.

Water scarcity is a pressing challenge in Maputo [48]. Hitherto, local authorities have followed the traditional approach of building dams as needed; however, if present water use trends continue, even these new sources will be insufficient by 2030 [49]. Moreover, access to safe potable water is problematic outside the city-centre, where more than half of the total population depends on groundwater supplied by small-scale independent water providers. In these areas high concentrations of nitrates (>250 mg/L, compared with the WHO safe limit of 50 mg/L) have been measured [50].

Less than 10 % of the population in Maputo is served by a sewer network, all in the city centre [51]. Moreover, only a fraction of the wastewater discharged into the network is conveyed to the existing STP, a pond system composed of two anaerobic and two facultative ponds, which is poorly maintained and ineffective. Informal and unsafe wastewater reclamation occurs on the premises of the STP; the plant is surrounded by farms (~120 ha) that supply some of the food consumed in Maputo, and local farmers collect water directly from the pond system to irrigate their crops.

Inhabitants of the peri-urban areas rely on on-site systems, with more than 50 % using latrines and another 40 % septic tanks [51]. Private operators are needed to empty these systems once they fill up and approximately 8 % of all faecal sludge is transported by truck to the STP and emptied directly into the anaerobic ponds, being “effectively treated” [52]. Part of the sludge is “safely abandoned” when the on-site systems are full (18 %) [50], although the aforementioned groundwater contamination with nitrates raises concerns about the safety of such disposal. A larger fraction (74 %) does not undergo safe treatment, and is likely “unsafely emptied” and left on the domestic premises when space is available [52].

Maputo’s current water system clearly encompasses numerous threats to the environment and to human health, with potential and actual knock-on effects on fishing, tourism, industrial development, and other economic sectors. One potential solution to diminish health risks and increase water availability lies in safe and regulated water reclamation for a variety of uses [53]. Water reclamation closes the urban water cycle, addressing water scarcity, environmental pollution and human-health issues. Informal unsafe water reclamation can be reduced, and, in addition, it can generate financial revenues: e.g., sludge can be transformed into fertilizers or used as fuel. These actions should positively impact the quality of the urban environment and ultimately lead to improved urban health. However, the existence of broad inter-linkages between sectors and strong feedback loops that regulate the behaviour of the water system imply that a systems analytic approach is needed to holistically understand these challenges and identify appropriate leverage points for action.

**Fig. 1:**
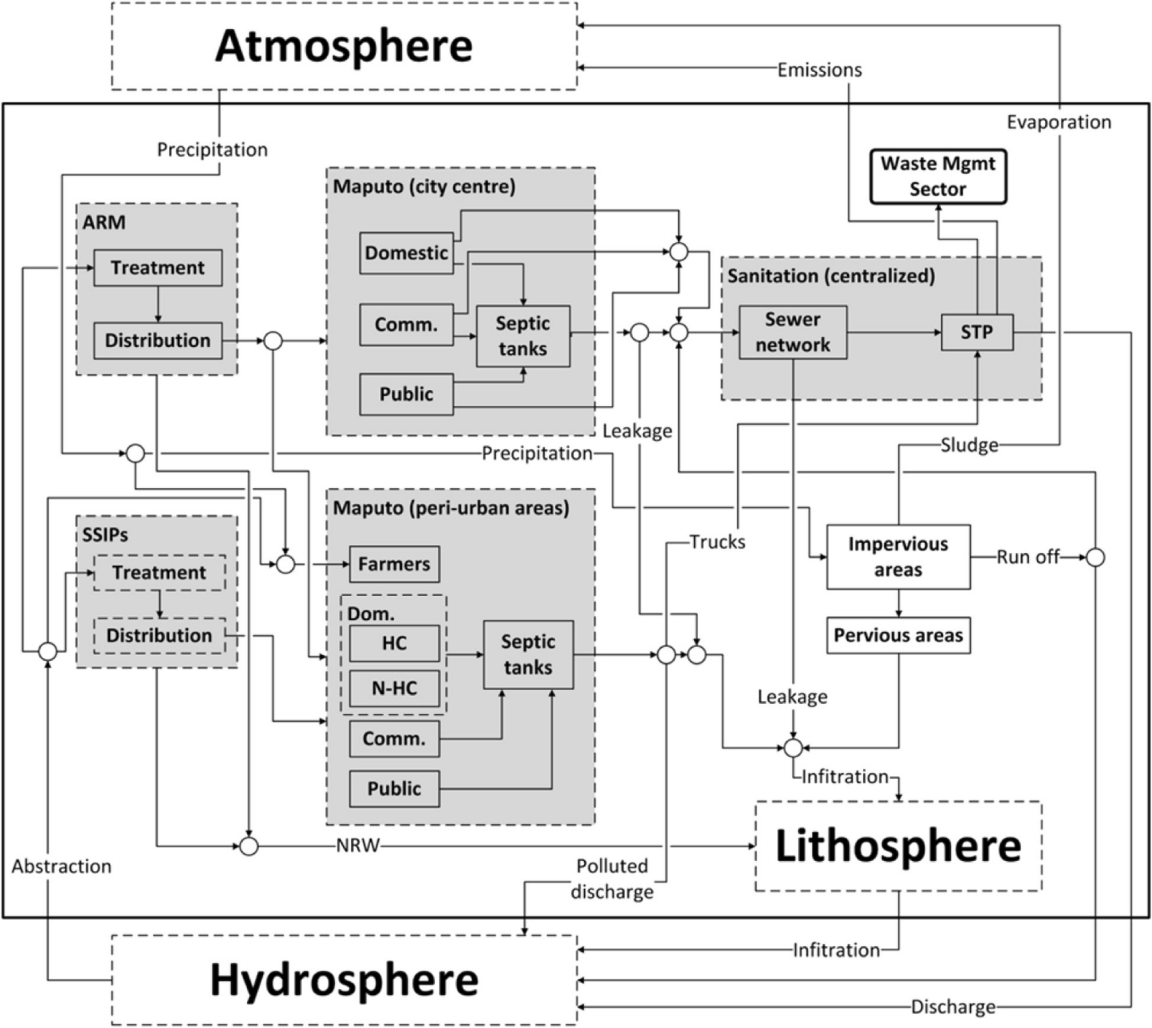
Material flow diagram for the existing water and sanitation sector in Maputo, adapted from [55]. Comm. = Commercial; NRW = non-revenue water; Dom. = Domestic; HC = human consumption; SSIPs = Small-scale independent (water) providers, these are private operators that supply water, from wells, to more than half of the population in Maputo; ARM = Águas da Região de Maputo, the largest water supplier in the city of Maputo; STP = Sewer treatment plant

A current project has taken such an approach, developing a conceptual mass flow analysis (MFA) (Fig. 1) visualizing flows of drinking water, wastewater, faecal sludge and rainwater in Maputo. MFA “is a systematic assessment of the flows and stocks of materials within a system defined in space and time” [54] and it allows for identification of linkages among different sectors in a system. This project will go on to quantify and map flows of energy, nutrients and health related vectors in the city, and will apply a cost-benefit analysis to further understand the implications of interventions in the field of water reclamation at different scales. These analyses should lead to substantially improved and policy-relevant evidence for decision-makers in Maputo.

#### Transdisciplinarity and a water mega-project in Vienna, Austria

A mega-project involving multi-sectoral water management in Vienna, Austria (population 1.6 million, 1971; 1.8 million, 2014) illuminates the potential benefits of transdisciplinary engagement in this context. Initially planned as a flood-control effort for the Danube River in Vienna, a series of government, expert and public consultations resulted in a much more ambitious project [56].

Flooding of the Danube, along with attendant loss of life and property, disruption of agriculture and viniculture, and spread of disease has historically been a major factor affecting Vienna. In the early 1970’s, the city administration, in collaboration with a set of consultants, decided to tackle this problem on a long-term basis; critically, this was seen as an opportunity to achieve more than flood control.

An interdisciplinary team of water resource engineers, architects, transportation and landscape planners, ecologists and limnologists, experts on waste water and pollution settled on a solution involving the creation of a second, parallel river to act as a flood channel, separated from the main channel by a 21 km long artificial island (the “Donauinsel”). In times of flooding resulting from seasonal melting of snow or heavy rainstorms, weirs are opened up to let in and contain flood waters before they enter the city, filling up the flood basin and reducing the flooding of cities and countries downstream. Indeed, Vienna is a notable exception to the severe flooding seen in European cities in recent years—from 2003 to 2009, 26 major flood disasters were recorded in Europe, and flooding over this period caused 320 deaths and EUR 17 billion in economic losses [55]. Whereas most cities can cope with flooding with a return period of 30–200 years (with Western countries generally on the higher end of the scale), Vienna is essentially permanently flood-safe, as “the Vienna flood protection system can manage flows with a return period of around 10 000 years” [56].

This project dealt with many aspects of urban water management beyond flood control. Prior to the project, raw sewage was expelled into the Danube, in both Vienna and in towns upstream. Sewage systems were introduced in the latter, while in Vienna 18 sewers were connected to a main trunk line on the banks of the river, carrying wastes to the new Euro Sewage Treatment Plant, globally recognized for innovative, treatment processes, and slated to become energy self-sufficient by 2020 [57]. In the new channel, complicated filtration systems were rejected in favor of natural filtration by the island itself, to reduce technological overkill and high maintenance costs. Phosphate and other fertilizer contents from upstream agriculture are thus filtered out by the land mass of the artificial island, allowing only clean water to pass into the new channel.

Through the project, Vienna, a landlocked city, came to enjoy a beachfront of 42 km, which now attracts ~300,000 people/day on summer weekends for recreational and sporting activities, some water-based—a huge positive contribution to physical and mental public health and family time. Green protected areas for recreation and wildlife conservation, including a new National Wetlands Park where native flora and fauna were reintroduced, added to the positive ecological balance of this huge engineering project. On the practical side, it opened up vast formerly flood-prone areas for commercial urban development, bringing in billions of Euros of revenue, greatly exceeding the sum spent on the project. New cross-river public transport train lines and a 120 MW hydroelectric plant integrated with the needed water table control barrier, a new rail-sea terminal for river-based barges, tourist boats, as well as schools, hotels, hospitals, parks and housing appeared in formerly unattractive and underused areas, contributing to the economy and increased employment.

The project faced significant opposition from its inception, partly political and partly arising from environmental concerns. This was met with a professionally-administered programme of public information, media work and stakeholder fora with progressive adaptation of details, culminating in a positive referendum for the project. Today, citizens take considerable pride in and ownership of the project, contributing to a multidimensional success story in urban water management [56].

## Conclusions

This review paper summarizes the strong and complex relationships between urban water management and health, which are a primary impetus for the use of integrated systems approaches in this context. Serious, concerted and localized urban water-oriented projects involving analytic systems methods; real engagement among scientists in different fields; the genuine involvement of stakeholders, including end-users, in intelligent processes; and interventions at multiple scales can achieve great strides in improving public health, economic activity, the environment, employment and the quality of life. This is shown via a set of case studies from different continents and a range of socio-economic contexts, which examine the complexity of the conjoined urban water and health system and provide examples of the benefits which can accrue from the application of elements of systems approaches.

Modelling of complexity in the water and wastewater cycles is illustrated in the case study of Maputo, where MFA gives insight as to probable demand for and sources of reclaimed wastewater. This allows for the identification of possible interventions to better manage water re-use and environmental pollution, and attendant health risks. Inter- and transdisciplinarity and multi-scale thinking and interventions are highlighted in all the case studies; increasing support from and involvement of stakeholders during the implementation phase of urban water management projects is noted as of particular importance. The involvement of street vendors and police officers in Kolkata was essential for the improvement of the street food system, leading to more jobs and improved environmental health. The research drew on insights from epidemiology, solid waste and water engineering, urban planning, and economics, and actions were taken at individual, community and city levels. The Surat case is a particularly good example of environmental and health co-benefits arising from effective water management. Moreover, it suggests that production of energy and fertilizer from wastewater can provide the impetus for small entrepreneurs to become involved in wastewater treatment, using waste as a source for their products. From the Maputo case study, we see that reclaimed water from the sanitation system can stimulate urban agriculture and industrial development in a sustainable way, while combating water scarcity, leading to better water supply and sanitation and thus improving urban health. This work explicitly considers multiple scales of intervention, from wastewater reclamation implemented at the individual level, which can benefit local industry or irrigation of gardens, to centralized systems that use effluent from the STP for larger-scale agricultural irrigation. Vienna provides a compelling example of transdisciplinary engagement leading to unanticipated benefits—citizens and many other stakeholders transformed a flood abatement project into a much broader effort, leading to new housing and business development and recreational opportunities, thus co-creating an integrated approach that yielded multiple benefits from an effort in environmental and urban protection. In this case, although actions were taken at the city level, a failure to consider how individuals would react to and benefit from the new land would significantly underestimate project benefits. In general, analysis and/or modelling of impacts, benefits and costs can provide evidence on the optimal scale or scales of intervention. A key element of the examples in this paper is the emphasis on the benefits of the interventions, in contrast to a focus on threats and costs.

None of the examples presented here involved a conscious effort to apply systems approaches as defined in this paper. A central assertion of this work is that such an effort would be of great utility in urban water management decisions to diminish health risks and environmental degradation. Some efforts have already been made in this direction, for example using system dynamics to predict municipal water demand in fast-growing urban areas where limited access to historical data complicates accurate prediction [58]; to study water resources management issues, and in particular policy decisions [59]; and to involve relevant stakeholders in decision-making processes regarding water savings [60]. The typical structure of both research and decision-making, however, is not oriented toward the adoption of systems approaches, but toward specialization and segregated authority, for practical reasons.

When systems approaches in urban water management are well executed, urban health can be improved with a minimum of additional direct costs, often leading to creative solutions, broad endorsement by stakeholders, and immense environmental and societal benefits. The question of how best to incentivize and catalyze such approaches and what systems methods should be used under different circumstances is important, and requires further research. In addition, further research is needed on the design, systematic implementation and evaluation of systems approaches in urban water management.

## Additional file

Additional file 1:
**Peer review reports.** (PDF 175 kb)
