# Supplementary material for: Improving health in cities through systems approaches for urban water management
Source: Environ Health. 2016 Mar 8;15(Suppl 1):31. doi: 10.1186/s12940-016-0107-2 (PMC4895287; doi:10.1186/s12940-016-0107-2)
Supplement: Additional file 1: — Peer review reports. (PDF 175 kb) [file 12940_2016_107_MOESM1_ESM.pdf]

Reviewer 1

I have reviewed the article. I find the case studies very interesting. However, I think that the overall narrative could be improved. Perhaps it is the term ”system approach” which is defined as a group of many different things which are not necessarily a system approach, e.g. last para on page 4 is what is actually known as “science-policy interface”. Mostly, the authors have tried to see the benefits gained to water management through other environmental management practices (sometimes even unintended) instead of a pre-conceived system approach.
Also the case studies often extend beyond water management and more as “water and sanitation” solutions, sometime even going much beyond waster, e.g. municipal waste which can also include solid waste and other type of wastes .

Just a clarification on second para p4, what kind of “mental and physical health opportunities” we are talking here, it remains unclear.

I don’t know if you have a formal feedback form which I need to fill in.
Best wishes,
shailendra
--
Shailendra Mudgal

Reviewer 2

Thank you again for inviting me for this review and my apologies for my (too) late reply.

- The paper is very interesting and gives a good insight in the benefits of systems approaches
- It is very well illustrated with examples and case studies
- It is a pity that the authors don't describe how an incentive can be created to use systems approaches in a more proactive preventive way instead of waiting for disasters to occur
- An interesting tool to measure the effectiveness of systems approaches would be the application of the City Blue Print methodology (see doi:10.1007/s11269-013-0462-5). I recommend that this is included in the discussion

Best regards,
Jan Hofman
